# Subacute Encephalopathy and Seizures in Alcoholics Syndrome

**DOI:** 10.31662/jmaj.2024-0248

**Published:** 2025-04-04

**Authors:** Rikuto Christopher Shinohara, Shun Takayanagi, Yoichi Furutaka, Shinya Watanabe

**Affiliations:** 1 Department of Psychiatry, National Hospital Organization Hokkaido Medical Center, Sapporo, Japan

**Keywords:** alcohol withdrawal, seizure, epilepsy

A 56-year-old male with alcohol dependence, abstinent for 2 days, was brought to the emergency department with generalized tonic-clonic seizures. He had no history of seizure or withdrawal and had discontinued outpatient treatment. Magnetic resonance imaging (MRI) showed focal hyperintensity (Figure 1A-C). Despite treatment with diazepam and lacosamide, he remained unconscious (E1V1M4). By day 4, his consciousness improved (E4V3M5). Electroencephalography (EEG) revealed lateralized periodic discharges (Figure 1D), confirming a diagnosis of subacute encephalopathy with seizures in alcoholics (SESA) syndrome. By day 24, he regained consciousness but had motor aphasia and visual hallucinations. By day 63, his symptoms remitted and he was transferred to rehabilitation (Figure 2A-D).

**Figure 1.**
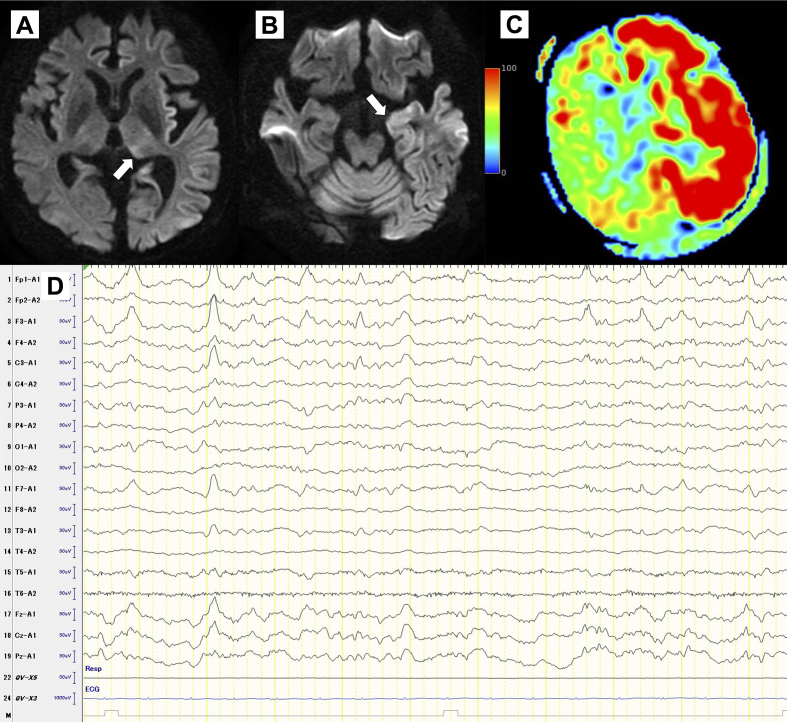
(A) and (B) Diffusion-weighted images (DWI) on initial presentation demonstrating increased signal intensity in the left pulvinar (A) and temporal tip (B). (C) Arterial spin labeling (ASL) on initial presentation demonstrated increased signal intensity in the left hemisphere. (D) Electroencephalography (EEG) on day 3, showed lateralized periodic discharges in the left frontal region.

**Figure 2.**
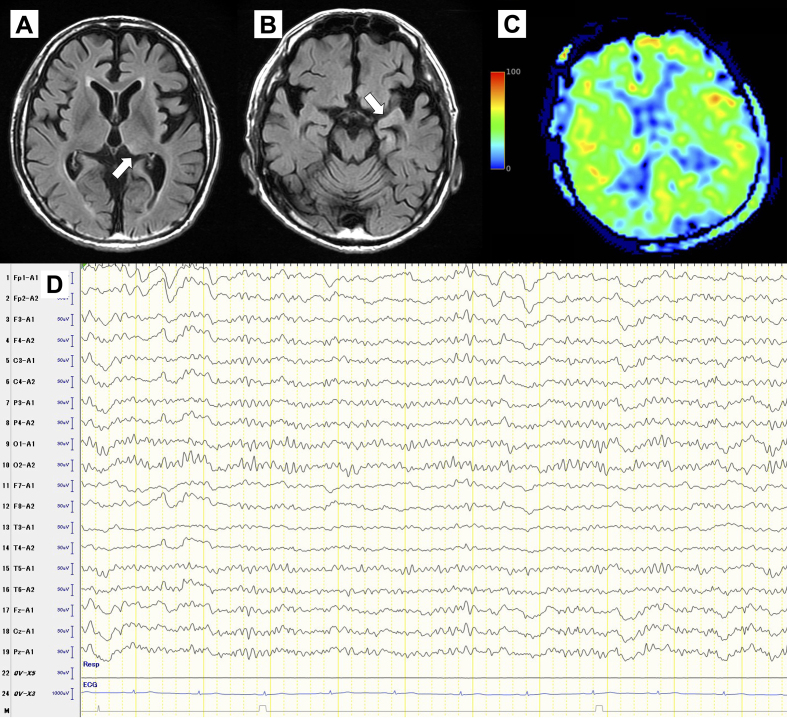
(A) and (B) Fluid-attenuated inversion recovery (FLAIR) on day 8, showing resolving hyperintensity in the left pulvinar (A) and temporal tip (B). (C) Arterial spin labeling (ASL) on day 8, demonstrated no increased signal intensity. (D) Electroencephalography (EEG) on Day 50, showed resolved lateralized periodic discharges.

SESA syndrome is a rare condition in alcohol-dependent individuals ^(1)^. Unlike typical withdrawal, it often involves prolonged confusion and recurrent seizures, requiring long-term antiepileptic treatment ^(2), (3)^. MRI frequently shows transient cortical-subcortical T2-hyperintense areas with restricted diffusion, and EEG reveals focal abnormalities ^(2), (4)^. The diagnosis of SESA syndrome is crucial due to its distinct course and management.

## Article Information

### Conflicts of Interest

None

### Acknowledgement

The authors thank Yudai Imahayashi MD, Keita Majima MD, Asuka Mori MD, Masayoshi Kawaguchi MD, and Akari Saratani MD for their cooperation in patient care.

### Author Contributions

Rikuto Christopher Shinohara contributed to patient care, planning, conduct, and writing up the work. Shun Takayanagi, Yoichi Furutaka, and Shinya Watanabe provided critical revisions to the report. All authors reviewed and approved the final version.

### Patient Consent

Consent to publish the details of the present case was obtained from the patient.
